# Catastrophizing and acceptance are mediators between insomnia and pain intensity—an SQRP study of more than 6,400 patients with non-malignant chronic pain conditions

**DOI:** 10.3389/fpain.2023.1244606

**Published:** 2023-09-27

**Authors:** Björn Gerdle, Elena Dragioti, Marcelo Rivano Fischer, Huan-Ji Dong, Åsa Ringqvist

**Affiliations:** ^1^ Pain and Rehabilitation Centre, Department of Health, Medicine and Caring Sciences, Linköping University, Linköping, Sweden; ^2^ Department of Neurosurgery and Pain Rehabilitation, Skåne University Hospital, Lund, Sweden; ^3^ Department of Health Sciences, Faculty of Medicine, Lund University, Lund, Sweden

**Keywords:** acceptance, anxiety, catastrophizing, depression, fear avoidance, insomnia, pain, physical activity

## Abstract

**Background:**

Sleep problems (insomnia) and chronic pain are associated. Chronic pain and insomnia/insufficient sleep quality share similar symptoms and features. Although they have a bidirectional relationship, more research is needed to understand how they interact via mediators and how moderators influence this relationship.

**Aims:**

In this large clinical registry-based cohort study (*N* = 6,497), we investigate important mediators between insomnia and pain intensity in a cross-sectional sample of chronic pain patients using advanced path analysis. In addition, we investigate whether some background variables were moderators of the identified important paths or not and the correlation patterns between insomnia and pain intensity in relation to the mediators.

**Methods:**

This study includes a cohort of adult patients with chronic non-cancer pain from the Swedish Quality Registry for Pain Rehabilitation (SQRP) with data on patient-reported outcome measures (PROMs) (2008–2016). The PROMs cover the background, pain aspects, psychological distress, pain-related cognitions, activity/participation, and health-related quality of life variables of the patients. Partial least squares structural equation modeling was used to explore the direct and indirect (via mediators) relationships between insomnia and pain intensity at baseline.

**Results:**

In this cohort study, insomnia was prevalent at 62.3%, and both direct and indirect mediating paths were present for the insomnia–pain intensity relationship. All of the mediating effects combined were weaker than the direct effect between insomnia and pain intensity. The mediating effects via catastrophizing and acceptance showed the strongest and equal mediating paths, and mediating effects via fear avoidance were the second strongest. Insomnia showed stronger direct significant correlations with psychological distress, catastrophizing, and acceptance compared with those of pain intensity. Sex, age, education level, spatial extent of pain, or body mass index did not moderate the mediating paths.

**Discussion and conclusion:**

This study confirms the existence of significant direct and mediating paths between reported insomnia and pain intensity. Future studies should focus on illuminating how sleep interventions influence pain intensity and other important key factors that contribute to the distress of chronic pain patients.

## 1. Introduction

Approximately 20% of the European adult population suffers from chronic pain of at least moderate intensity (1). Chronic pain patients frequently report comorbidities, increased sick leave, and low quality of health (2). These common comorbidities include obesity, insomnia, cardiovascular conditions, and anxiety and depressive symptoms (2, 3). Insomnia, the most prevalent sleep disorder, is estimated to have a prevalence rate of 10%–15% in adults (4, 5). Insomnia/poor quality of sleep is associated with an increase in light sleep and a decrease in the deepest sleep stages (i.e., slow-wave sleep) (6, 7). Prevalence rates in cohorts of chronic pain patients differ considerably depending on the measures used. However, a recent systematic review (SR) of studies using the Insomnia Severity Index (ISI) reported a prevalence of 72.9% in chronic pain patients (8), indicating that the prevalence of insomnia is considerably higher in chronic pain conditions.

Several studies, including polysomnography studies, have reported cross-sectional and longitudinal associations between sleep problems/insomnia and chronic pain (6, 7, 9–15). Thus, poor sleep increases pain and pain affects sleep. Longitudinal studies reported that poor sleep is the factor with the strongest empirical support for its effect on chronic pain (14, 16, 17). Moreover, a large prospective study conducted over 11 years found that obesity, poor sleep, and chronic disease all predicted persistent chronic widespread pain (CWP), except for depression (15). In addition, the number of sleep-related complaints was found to be positively and substantially correlated with an increased risk of developing CWP, musculoskeletal pain, and pain-related disability (18). Thus, insomnia seems closely connected to the development of chronic pain.

Chronic pain and insomnia/insufficient sleep quality share similar symptoms and features, including age (1, 19), obesity (20, 21), female sex (1, 22), catastrophizing (23, 24, 25), low physical activity (PA) (26, 27), socioeconomic factors such as education (3, 22), and anxiety and depressive symptoms (28–32). In addition, the literature suggests a possible role of acceptance both for chronic pain and insomnia (33, 34). Hence, a link between sleep and pain is well established, but the mechanisms for their associations—for instance, the relative importance of direct associations and mediating factors—are less well understood, although polymodal influences of sleep on pain have been proposed. Impaired sleep is believed to have a negative influence on top-down pain control and sensitization and lower the capacity to adequately attend to or disengage from pain—i.e., a reduced allocation of attentional resources (35, 36). Other possible shared mechanisms for pain and insomnia and possibly mediating factors that are discussed include affect and mood, catastrophizing, endogenous pain modulation including activated hyperalgesic systems, mesolimbic dopaminergic pathways and serotonergic pathways, low-grade inflammatory substances and other endogenous substances [e.g., growth hormone (GH) and prolactin], alterations in autonomic balance, and a cyclic alternating pattern (6, 7, 37–39).

More research is needed to determine whether mediators are present between insomnia and chronic pain or not (40–42). A mediator, located between the independent and the dependent variable, can either increase or decrease the effect of the independent variable. A SR identified nine cross-sectional mediation studies focusing on the sleep–pain relationship (43). Although there was some evidence for the mediating roles of the psychological and physiological aspects of emotional and attentional processes, methodological limitations remained apparent (43). Furthermore, the studies included were generally small; only one study included more than 300 chronic pain patients. Hence, large cohort studies are required to investigate the mediators for the insomnia–pain intensity relationships in chronic pain patients. Although longitudinal studies of mediators are needed (43), cross-sectional studies are also important for understanding the clinical presentations of patients during assessment. Considering the available data from the Swedish Quality Registry for Pain Rehabilitation (SQRP) and the referred results from the literature, we found that it is possible to investigate the importance of the following five mediators, namely, catastrophizing, fear avoidance, physical activity, acceptance, and psychological distress, for insomnia–pain intensity relationship.

In addition, important moderators of direct and mediation paths between insomnia and pain aspects in chronic pain patients must also be identified. A moderator effect is present when a causal relationship between two variables changes due to a change in a third variable, which indicates cohort heterogeneity. Common examples of moderators are sex, age, and education level.

Cross-sectional studies are important for understanding the clinical presentations of patients. This cross-sectional study of chronic pain patients employs advanced path analysis to investigate mediators between insomnia and pain intensity using data from a large clinical registry-based cohort. In line with this, we investigate whether the five variables—sex, age, obesity, education level, and spatial extent of pain—are moderators of the identified paths or not and the correlation patterns between insomnia and pain intensity in relation to the mediators.

## 2. Subjects and methods

### 2.1. Subjects and the Swedish Quality Registry for Pain Rehabilitation

Chronic pain patients from most specialist departments in Sweden report patient-reported outcome measures (PROMs) to the SQRP (44). A cohort of adult patients (i.e., ≥18 years) who had chronic non-malignant pain and were registered in the SQRP between 2008 and 2016 was included. The pain conditions are generally complex, i.e., comorbidities are prevalent, prolonged sick leave, inadequate coping, and/or unimodal treatment failures. This clinical registry does not have strict inclusion and exclusion criteria. Patients with severe psychiatric morbidity, a history of drug and alcohol abuse, and conditions that do not allow physical exercise are excluded. Swedish guidelines, which have been approved by several authorities and professional organizations, recommend that interdisciplinary pain rehabilitation programs (IPRPs) is offered to chronic pain patients with complex clinical presentations, including insufficient coping strategies, and when monodisciplinary interventions have failed (45). Most primary care physicians refer patients to specialist care, and no exact figures are available regarding the number of patients managed within primary care with monodisciplinary pain interventions.

### 2.2. Ethics

This study was conducted in accordance with the Helsinki Declaration. The Ethical Review Board in Linköping (Dnr: 2015/108-31) approved the study. All included patients gave their written consent after written information about the study. We did not exactly know how many patients declined to participate in the SQRP. Overall estimations made by the steering group of the SQRP indicated that more than 90% of the patients referred to the specialist departments in Sweden choose to participate in the SQRP.

### 2.3. PROMs

The PROMs cover sociodemographic characteristics, pain, psychological variables, cognitions, participation factors, and quality of life facets. The patients completed the PROMs up to three times. This study used baseline data, and we only provided a brief description of the variables because they have been described in detail in previous studies (2, 46–48).

### 2.4. Background variables

The following aspects were used:

• Age (years).
• Sex.
• Education level (university education versus no university education).
• Country of birth (in versus outside of Europe).
• Number of days with pain.
• Number of painful areas (range: 1–36) [pain region index (PRI)] (2, 49).
• Body mass index (BMI, kg/m^2^) was determined. The World Health Organization (WHO) criteria were used for classification: <18.5 kg/m^2^ = underweight; 18.5–24.9 kg/m^2^ = normal; 25.0–29.9 kg/m^2^ = overweight; ≥30.0 kg/m^2^ = obese.

#### 2.4.1. Path model analysis

**Figure 1:**
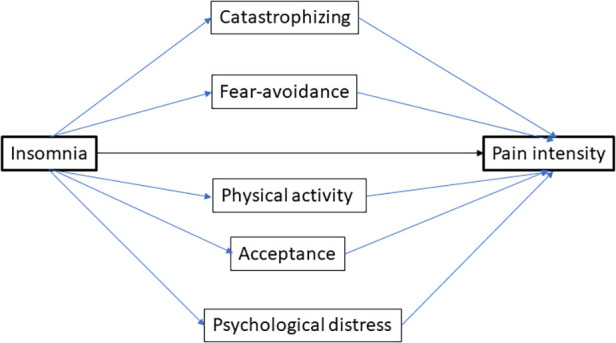
The theoretical model of the Insomnia–Pain intensity relationship together with five mediating paths. Latent variables (constructs) are shown together with the paths and directions. Note that a mediating path consists of two parts—i.e., from insomnia to the moderator (part 1) and from the mediator to pain intensity (part 2). The standardized coefficient *β* for the mediating path is obtained by multiplying the standard coefficients for the two parts of the mediating path.

Consistent with the recent SR, we investigated the mediation paths for the insomnia–pain intensity relationship (43). Throughout the text below, we capitalize the initial letter of latent variables (constructs)—e.g., Insomnia and Pain intensity. Five mediation paths were investigated, i.e., 1) Catastrophizing, 2) Fear avoidance, 3) Physical activity level, 4) Acceptance and 5) Psychological distress (Figure 1). The arrows in the figures and text (–>) express a hypothesized relationship in agreement with the referred SR (43).

### 2.5. Variables included in the path analyses

#### 2.5.1. Pain intensity aspects

Three variables were used as indicators of Pain intensity, namely, (1) NRS-7d, average pain intensity over the past week using a numerical rating scale (0 = no pain and 10 = worst possible pain); (2) MPI-pain severity, pain severity scale (range: 0–6) of the multidimensional pain inventory (MPI) that indicates current and average pain intensity over the previous week; and (3) RAND36-bodily pain, the bodily pain subscale (range: 0–100) of the Short Form Health Survey (SF36), where a low value denotes a high intensity. Two identical versions of the SF36 exist; the license-free RAND36 was used.

#### 2.5.2. Psychological distress aspects

Four variables were included as indicators: (1) HADS-tot, the two scales of the hospital anxiety and depression scale (HADS), which obtain symptoms of depression and anxiety (50, 51), were summed (range: 0–42) (52); (2) MPI-distress, this subscale of MPI captures the perceived feelings of patients concerning anxiety, depression, and irritation in the previous week (range: 0–6); (3) RAND36-mental health, based on items concerning mood, mental wellbeing, and behavioral/emotional control, with a range of 0–100, where a high value denotes good mental health; and (4) RAND36-role emotional, a subscale based on three items covering the role limitations related to emotional facets, with a range of 0–100, where a high value denotes a positive situation.

#### 2.5.3. Fear avoidance aspects

The Tampa scale for kinesiophobia (TSK) (range: 17–68) was used because it is a validated scale used to indicate fear avoidance—i.e., fear of injury or (re)injury—in cohorts of chronic pain patients (53–57).

#### 2.5.4. Acceptance aspects

To indicate acceptance, we used the validated Swedish version of the chronic pain acceptance questionnaire eight-item version (CPAQ8) (58). This questionnaire consists of two subscales: the activity engagement scale (CPAQ8-AE) (range: 0–24) and the pain willingness scale (CPAQ8-PW) (range: 0–24) (59). In the literature, the total sum of CPAQ8 is used as a general indicator of acceptance (CPAQ8-tot) (range: 0–48).

#### 2.5.5. Catastrophizing aspects

The pain catastrophizing scale (PCS) was used to map catastrophizing aspects. It measures three dimensions of pain catastrophizing: rumination (PCS-rum) (range: 0–16); helplessness (PCS-help) (range: 0–24); and magnification (PCS-Magn) (range: 0–12) (60–62). These three scales represent different cognitive processes. Rumination is a tendency to have repetitive thoughts and dwell on pain and its impact. Helplessness indicates a reduced confidence in the ability to cope with the pain. Magnification is the tendency to overemphasize the seriousness or threat of pain.

#### 2.5.6. Physical activity-level aspect

Physical activity level was assessed using a question about physical activity developed by the Swedish National Board of Health and Welfare. This ordinal item has the following wording: “How much time do you spend in a typical week doing physical exercise that leaves you out of breath, such as running, calisthenics or ball sports?” (63). The respondents chose one of the following: 0, <30, 30–60, 60–90, 90–120, or >120 min.

#### 2.5.7. Insomnia aspects

The ISI captures the degree of insomnia symptoms (64). The scores of seven items (range: 0–4) are summed to produce the total ISI score (range: 0–28), which is divided into four categories; 0–7 = no insomnia; 8–14 = sub-threshold insomnia; 15–21 = moderate insomnia; and 22–28 = severe insomnia. A score of ≥15 was deemed as clinical insomnia.

#### 2.5.8. Moderators

Sex/gender, education level, age, PRI, and BMI were investigated as possible moderators. All moderators were coded as binary/categorical variables. For education, the levels of university versus no university were applied. For age and PRI, the median values were used as cutoffs. For BMI, ≥30 kg/m^2^ (i.e., obesity) was used as a cutoff.

### 2.6. Statistics

The statistical packages IBM SPSS Statistics (version 28.0; IBM Corporation, Route 100 Somers, NY, USA) and SmartPLS version 4 (SmartPLS 4, Boenningstedt, SmartPLS. Retrieved from https://www.smartpls.com) were used. The mean values (±1 standard deviation; SD) of continuous variables and percentages (%) for categorical variables are presented. For the coefficients of the path analyses, mean ± SD, *t*-values, *p*-values, and 95% CI are reported. The retrieved data set included missing data (Table 1). Due to the low percentages of missing data, these were replaced with the mean values in the path analyses.

**Table 1:** Descriptive data for the included cohort of chronic pain patients: mean, 1 standard deviation (SD), and % missing data.

Variables	Mean	SD	% missing data
Age	43.02	12.76	0.00
BMI	27.23	5.66	0.10
PRI	15.42	8.72	0.00
ISI	16.35	6.73	0.00
NRS-7d	6.91	1.72	0.80
RAND36-bodily pain-rev	75.22	15.68	1.40
MPI-pain severity	4.18	1.03	0.20
PCS-tot^a^	27.02	11.03	0.10
PCS-rum	9.08	4.10	0.00
PCS-help	13.15	5.48	0.10
PCS-Magn	4.79	2.94	0.10
TSK	38.93	8.91	0.00
Exercise	2.40	1.65	1.20
CPAQ8-tot	19.86	8.50	0.00
CPAQ8-AE^a^	10.70	5.57	0.00
CPAQ8-PW^a^	9.16	5.01	0.00
HADS-tot	18.07	8.34	0.40
HADS-A^a^	9.11	4.79	0.40
HADS-D^a^	8.96	4.52	0.40
MPI-distress	3.48	1.32	0.30
RAND36-mental health-rev	44.78	21.27	0.30
RAND-role emotional-rev	57.31	42.16	1.20

-rev, the variable was revised to indicate a troublesome situation; NRS-7d, pain intensity according to a numeric rating scale; HADS, hospital anxiety and depression scale; HADS-tot, sum of the two subscales of the HADS; MPI, multidimensional pain inventory; RAND36, the free version of the Short Form Health Survey (SF36); TSK, Tampa scale for kinesiophobia; CPAQ, chronic pain acceptance questionnaire eight-item version; CPAQ8-AE, activity engagement subscale of CPAQ; CPAQ-PW PA, pain willingness subscale of CPAQ; physical activity level; ISI, insomnia severity index; PCS, pain catastrophizing scale; PCS-rum, rumination subscale of PCS; PCS-help, helplessness subscale of PCS; PCS-Magn, magnification subscale of PCS; university, binary variable measuring education level (university education vs. other education levels); PRI, pain region index; BMI, body mass index.

^a^ Indicates that the variable not was included in the final PLS-SEM analysis.

SmartPLS version 4 was used for the partial least squares structural equation modeling (PLS-SEM). This is a non-parametric method that models and estimates complex relationships among variables [see (65) for details including performance recommendations]. Generally, several indicators were used to define a latent variable (construct), and these needed to have the same direction (i.e., positively intercorrelated). Therefore, some indicators were reversed. RAND36-bodily pain can illustrate this. The scale has a possible range of 0–100, where a high value means low pain intensity and a low value means high pain intensity. To be consistent with other scales that capture the intensity of pain, i.e., NRS-7 days and MPI-pain severity where high values mean high pain intensity, the following calculation was made: 100—RAND36-bodily pain = RAND36-bodily pain-reversed. Using loadings, the outer model describes the relationships between the latent variable and their indicator variables. A reflective relationship was assumed for all latent variable–indicator relationships (not relevant for single-indicator latent variables). The inner model displayed the associations (paths) between the defined latent variables.

#### 2.6.1. Evaluation of the outer model

A close association (*indicator reliability*) between the indicators and the latent variables was necessary. For each indicator, the loadings were used to determine if such an association is present or not. For indicator reliability, the absolute outer loadings of >0.708 (possible range: −1 to +1) were required (65). Indicators were excluded when absolute loadings ≤0.40. For indicators with loadings 0.40–0.708, the exclusion was made if internal consistency and convergent validity increased. The composite reliability coefficient (rho_c_) (range: 0–1, with >0.50 required) was used to measure the *internal consistency reliability*. The average variance extracted (AVE) (range: 0–1, with >0.50 required) was used to measure the *convergent validity*. The *discriminant validity* was indicated using the heterotrait–monotrait ratio (HTMT) (values <0.90 required, preferably <0.85).

#### 2.6.2. Evaluation of the inner model

To check the *collinearity*, we used the variance inflation factor (VIF); values <5 were required (65). To determine path coefficients (standardized; *β*) including specific indirect (mediating paths) effects, we applied the bootstrapping technique. The following specifications were used for the bootstrapping: complete bootstrapping, percentile bootstrap, 10,000 samples, and two-tailed (*p* = 0.05). The determination coefficient (*R*^2^) (range: 0–1) indicating *explanatory power* and the effect size (*f*^2^) were also determined. The latter was used to indicate clinical relevance: <0.02 = no measurable effect; 0.02–0.14 = small effect; 0.15–0.34 = medium effect; and ≥0.35 large effect (65, 66). The mean ± SD, *t*-values, *p*-values, and 95% CI of these coefficients were obtained from the bootstrapping.

For mediating effect sizes (i.e., clinical importance), we applied the following guidelines (66, 67): *β*, 0.01–0.08 = small effect; 0.09–0.24 = medium effect; and ≥0.25 = large effect.

When analyzing the possible effects of the five moderators, we focused on the direct effect of insomnia on pain intensity and the most important mediator paths. All moderators were transformed into binary variables (see above), and multigroup analysis (MGA) was conducted to analyze moderator effects (65).

While *predictive power* was not the focus of this study, we determined *Q*^2^_predictive_ values (>0 indicated predictive relevance). The greater the *Q*^2^, the greater the predictability (68).

## 3. Results

### 3.1. The investigated cohort

The investigated cohort consisted of 6,497 patients, of whom 74.6% were women. One-third (31.2%) had the highest education level (i.e., university education). Descriptive data for the continuous variables are shown in Table 1. Moderate or severe insomnia was reported by 62.3% of the patients. The mean pain intensity according to NRS-7d was moderate to high (mean = 6.9, SD = 1.7). The proportion of obese patients was 27.4%. The proportions of definite cases of anxiety and depression (cutoff ≥ 11 for both subscales) according to HADS were 38.7% and 37.4%, respectively; in addition, 48.2% reported no signs of definite anxiety or depression.

### 3.2. PLS-SEM

#### 3.2.1. Evaluation of the outer model

**Figure 2:**
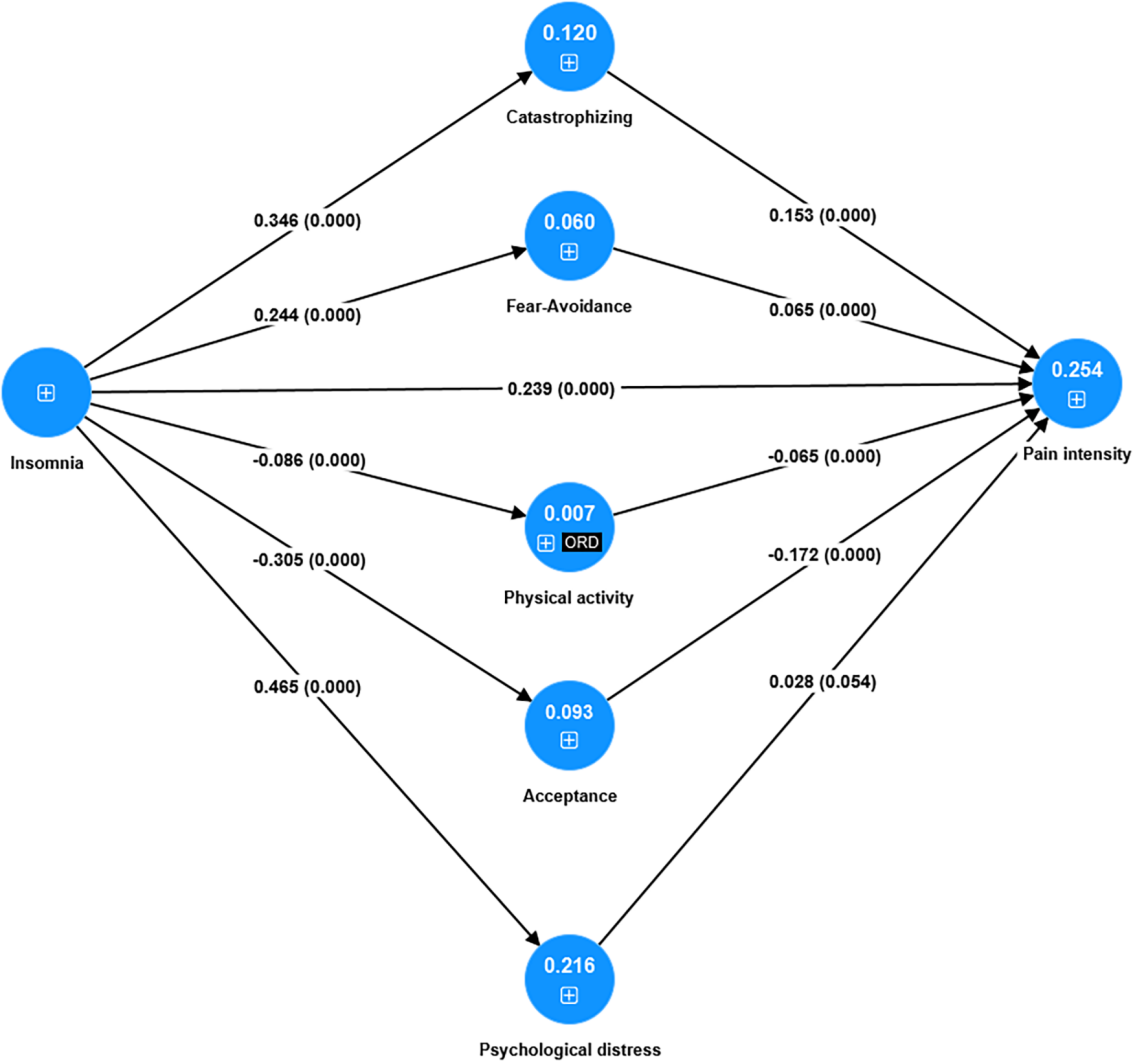
The final model analyzed using PLS-SEM (*N* = 6,497), that is, if insomnia affects pain intensity together with the five mediating paths. The blue circles show the latent variables (constructs). Loadings are not shown (see Table 3). For each path, the path coefficient *β* and the *p*-value are in parentheses (0.000 denotes *p* < 0.001). The explained variance (*R*^2^) is reported within the relevant latent variables. ORD = ordinal indicator for this latent variable.+sign indicates the existence of indicator/s for the latent variable.

The included indicators in the subsequent PLS-SEM analyses and their latent variables are listed in Table 2. In the initial analysis indicator, reliability (i.e., outer loadings) was above the threshold (i.e., above 0.708) for all indicators except for one of the subscales of CPAQ8. Internal consistency reliability (rho_c_) and convergent validity (AVE) increased and were both above the >0.50 level when the sum of the two CPAQ8 subscales (i.e., CPAQ8-tot) was used instead. Hence, Acceptance only had one indicator. The final model is shown in Figure 2, loadings in Table 3, and internal consistency reliability (rho_c_: 0.906–0.925) and convergent validity (AVE: 0.755–0.772) in Supplementary Table S1. The discriminant validity was satisfactory (well below 0.85) for all relevant latent variable combinations according to HTMT (Supplementary Table S2). In conclusion, the final outer model was associated with good indicator reliability, internal consistency reliability, convergent validity, and discriminant validity.

**Table 2:** List of the latent variables (constructs) and their potential indicators together with the moderators included in the PLS-SEM analyses.

Latent variable (construct)	Indicators
Insomnia	ISI
Pain intensity	NRS-7d MPI-pain severity RAND36-bodily pain-rev
Catastrophizing	PCS-rum PCS-help PCS-Magn
Fear avoidance	TSK
Physical activity	PA level
Acceptance	CPAQ8-AE and CPAQ8-PW or CPAQ8-tot
Psychological distress	HADS-tot MPI-distress RAND36-mental health-rev RAND36-role emotional-rev
Moderators	
Sex	Sex
Education level	University
Age	Age
Spatial extent of pain	PRI
Body Mass	BMI

-rev, the variable was revised to indicate a troublesome situation; NRS-7d, pain intensity according to a numeric rating scale; HADS, the hospital anxiety and depression scale; HADS-tot, sum of the two subscales of HADS; MPI, multidimensional pain inventory; RAND36, the free version of the Short Form Health Survey (SF36); TSK, Tampa scale for kinesiophobia; CPAQ, chronic pain acceptance questionnaire eight-item version; CPAQ8-AE, activity engagement subscale of CPAQ; CPAQ-PW, pain willingness subscale of CPAQ; ISI, insomnia severity index; PA, physical activity; PCS, pain catastrophizing scale; PCS-rum, rumination subscale of PCS; PCS-help, helplessness subscale of PCS; PCS-Magn, magnification subscale of PCS; university, binary variable measuring education level (university education vs. other education levels); PRI, pain region index; BMI, body mass index.

**Table 3:** Loadings of the indicators in the final model (cf. Figure 2). Note that when a latent variable has one indicator, the loading is 1.0.

Latent variable	Indicator	Loading
Insomnia	ISI	1.000
Pain intensity	NRS-7d	0.881
Pain intensity	RAND36-bodily pain-rev	0.854
Pain intensity	MPI-pain severity	0.901
Catastrophizing	PCS-help	0.920
Catastrophizing	PCS-Magn	0.849
Catastrophizing	PCS-rum	0.849
Fear avoidance	TSK	1.000
Physical activity	Exercise	1.000
Acceptance	CPAQ8-tot	1.000
Psychological distress	HADS-tot	0.911
Psychological distress	MPI-distress	0.882
Psychological distress	RAND36-mental health-rev	0.917
Psychological distress	RAND36-role emotional-rev	0.756

-rev, the variable was revised to indicate a troublesome situation; NRS-7d, pain intensity according to a numeric rating scale; HADS, the hospital anxiety and depression scale; HADS-tot, sum of the two subscales of HADS; MPI, multidimensional pain inventory; RAND36, the free version of the Short Form Health Survey (SF36); TSK, Tampa scale for kinesiophobia; CPAQ8-tot, chronic pain acceptance questionnaire eight-item version-total score; ISI, insomnia severity index; PCS, pain catastrophizing scale; PCS-rum, rumination subscale of PCS; PCS-help, helplessness subscale of PCS; PCS-Magn, magnification subscale of PCS.

^a^ Indicates that the variable not was included in the final PLS-SEM analysis.

#### 3.2.2. Evaluation of the inner model

The VIF values were below 3.70 (1.00–3.66)—i.e., model collinearity was not an issue. For details, see Supplementary Table S3.

The path coefficients (*β*) are presented in Figure 2 and Table 4. All path coefficients (direct effects), except between Psychological distress and Pain intensity (*p* = 0.054), were highly significant (all other *p*-values <0.001).

**Table 4:** Path coefficients (*β*) for the PLS-SEM model as shown in Figure 2. The mean ± SD, *t*-values, *p*-values, and 95% CI are shown.

Path	Mean	SD	*t*-statistics	*p*-values	95% CI lower	95% CI upper
Insomnia –> Pain intensity	0.239	0.012	19.159	<0.001	0.215	0.263
Insomnia –> Catastrophizing	0.346	0.011	31.759	<0.001	0.325	0.368
Insomnia –> Fear avoidance	0.245	0.012	20.826	<0.001	0.222	0.268
Insomnia –> Physical activity	−0.086	0.013	6.841	<0.001	−0.111	−0.062
Insomnia –> Acceptance	−0.305	0.012	26.200	<0.001	−0.327	−0.282
Insomnia –> Psychological distress	0.465	0.010	47.520	<0.001	0.446	0.484
Catastrophizing –> Pain intensity	0.154	0.016	9.671	<0.001	0.123	0.185
Fear avoidance –> Pain intensity	0.065	0.013	4.873	<0.001	0.039	0.091
Physical activity –> Pain intensity	−0.065	0.011	5.746	<0.001	−0.087	−0.043
Acceptance –> Pain intensity	−0.172	0.014	12.278	<0.001	−0.200	−0.145
Psychological distress –> Pain intensity	0.028	0.015	1.928	0.054	−0.000	0.056

The model in Figure 2 explained 25% of the variation in Pain intensity—i.e., *R*^2 ^= 0.254 ± 0.010, *p* < 0.001. Insomnia showed the strongest absolute direct effects with Psychological distress (*β* = 0.465 ± 0.010, *p* < 0.001; medium effect size according to *f*^2^), Catastrophizing (*β* = 0.346 ± 0.011, *p* < 0.001; small effect size according to *f*^2^), Acceptance (*β* = −0.305 ± 0.012, *p* < 0.001, small effect size according to *f*^2^), and Fear avoidance (*β* = −0.245 ± 0.012, *p* < 0.001, small effect size according to *f*^2^). Hence, the relationships between Insomnia and Pain intensity (*β* = 0.239 ± 0.012, *p* < 0.001; small effect size according to *f*^2^) and between Insomnia and Physical activity (*β* = -0.086 ± 0.013, *p* < 0.001; non-significant effect size) were weaker according to the path coefficients. The coefficients of determination (*R*^2^) showed similar patterns as the path coefficients—i.e., Psychological distress (*R*^2 ^= 0.216 ± 0.009), Catastrophizing (*R*^2 ^= 0.120 ± 0.008), and Acceptance (*R*^2 ^= 0.093 ± 0.007). For details, see Table 5. The effect sizes according to *f*^2^ are shown in Table 6. In conclusion, Insomnia showed significant direct associations with Pain intensity and the five mediators.

**Table 5:** The coefficients of determination (*R*^2^) for the relevant latent variables in the PLS-SEM model as depicted in Figure 2. The mean ± SD, *t*-values, *p*-values, and 95% CI are shown.

Latent variable	Mean	SD	*t*-statistics	*p*-values	95% CI lower	95% CI upper
Pain intensity	0.254	0.010	26.610	<0.001	0.237	0.274
Catastrophizing	0.120	0.008	15.874	<0.001	0.105	0.135
Fear avoidance	0.060	0.006	10.403	<0.001	0.049	0.072
Physical activity	0.007	0.002	3.391	0.001	0.004	0.012
Acceptance	0.093	0.007	13.109	<0.001	0.080	0.107
Psychological distress	0.216	0.009	23.756	<0.001	0.199	0.234

**Table 6:** Effects sizes according to *f*^2^ for the paths in the PLS-SEM model in Figure 2. The mean ± SD, *t*-values, *p*-values, and 95% CI are shown together with the clinical importance (see Methods for details concerning the categorization).

Paths	Mean	SD	*t*-statistics	*p*-values	95% CI lower	95% CI upper	Clinical importance
Insomnia –> Pain intensity	0.059	0.006	9.201	<0.001	0.047	0.072	Small
Insomnia –> Catastrophizing	0.137	0.010	13.951	<0.001	0.118	0.156	Small
Insomnia –> Fear avoidance	0.064	0.007	9.768	<0.001	0.052	0.077	Small
Insomnia –> Physical activity	0.008	0.002	3.361	0.001	0.004	0.012	No effect
Insomnia –> Acceptance	0.103	0.009	11.876	<0.001	0.086	0.120	Small
Insomnia –> Psychological distress	0.276	0.015	18.593	<0.001	0.248	0.306	Medium
Catastrophizing –> Pain intensity	0.016	0.003	4.726	<0.001	0.010	0.023	No effect
Fear avoidance –> Pain intensity	0.004	0.001	2.413	0.016	0.001	0.007	No effect
Physical activity –> Pain intensity	0.005	0.002	2.834	0.005	0.002	0.010	No effect
Acceptance –> Pain intensity	0.025	0.004	6.038	<0.001	0.017	0.033	Small
Psychological distress –> Pain intensity	0.001	0.001	0.894	0.371	0.000	0.002	No effect

**Table 7:** Specific indirect effects (i.e., mediating effects *β*). The mean ± SD, *t*-values, *p*-values, and 95% CI are shown together with the clinical importance (see Methods for details concerning the categorization).

Mediating paths	Mean	SD	*t*-statistics	*p*-values	95% CI lower	95% CI upper	Clinical importance
Insomnia –> Catastrophizing –> Pain intensity	0.053	0.006	9.281	<0.001	0.042	0.065	Small
Insomnia –> Fear avoidance –> Pain intensity	0.016	0.003	4.693	<0.001	0.009	0.023	Small
Insomnia –> Physical activity –> Pain intensity	0.006	0.001	4.316	<0.001	0.003	0.008	No effect
Insomnia –> Acceptance –> Pain intensity	0.053	0.005	10.987	<0.001	0.043	0.062	Small
Insomnia –> Psychological distress –> Pain intensity	0.013	0.007	1.924	0.054	−0.000	0.026	NA

NA,  not applicable.

Generally, weaker but still significant direct associations (paths) were observed for the direct relationships between each mediator and Pain intensity (Table 4). However, the clinical importance was limited because the effect sizes were non-significant for four of the paths and small for the Acceptance–Pain intensity relationship.

#### 3.2.3. Mediating effects (special indirect effects)

The total indirect effects (all mediating effects taken together) (*β* = 0.140 ± 0.007, *p* < 0.001; small effect size) were weaker than the direct path coefficient between Insomnia and Pain intensity (*β* = 0.239 ± 0.012, *p* < 0.001; medium effect size).

All of the mediating effects investigated except for Psychological distress (i.e., insomnia –> psychological distress –> pain intensity; *p* = 0.054) were significant (*p*-values < 0.001) (Table 6). The strongest and equal mediating effects were noted for Catastrophizing (*β* = 0.053 ± 0.006, *p* < 0.001; small effect size) and Acceptance (*β* = 0.053 ± 0.005, *p* < 0.001; small effect size), which were followed by Fear avoidance (0.016 ± 0.003, *p* < 0.001; small effect size) (Table 6). Also, the other significant mediating paths had small effect sizes except those involving Physical activity, which was associated with a non-significant effect size.

#### 3.2.4. Predictive power

The model had predictive relevance according to the *Q*^2^_predictive_ values (i.e., all > 0.00) (Supplementary Table S4).

#### 3.2.5. Moderating effects

None of the mediating paths was significantly influenced by any of the five moderators, but other effects were found for four of the moderators.

##### 3.2.5.1. Sex

No significant path coefficient differences were found between men and women.

##### 3.2.5.2. Age

Age was associated with four significant group differences:

(1) Insomnia –> Pain intensity (group difference: *p* > 0.001; younger: 0.273 ± 0.017 *p* < 0.001 vs. older: 0.185 ± 0.018 *p* < 0.001);
(2) Insomnia –> Catastrophizing (group difference: *p* = 0.033; younger: 0.331 ± 0.016 *p* < 0.001 vs. older: 0.377 ± 0.015 *p* < 0.001);
(3) Insomnia –> Fear avoidance (group difference: *p* = 0.045; younger: 0.225 ± 0.017 *p* < 0.001 vs. older: 0.272 ± 0.016 *p* < 0.001); and
(4) Insomnia –> Psychological distress (group difference: *p* = 0.013; younger: 0.451 ± 0.014 *p* < 0.001 vs. older: 0.499 ± 0.013 *p* < 0.001).

##### 3.2.5.3. Education level

One of the paths differed between those without and with university education (Group difference: *p* = 0.007)—i.e., Acceptance –> Pain intensity without university education (−0.149 ± 0.017 95% CI: −0.182 to −0.117) vs. Acceptance –> Pain intensity with university education (−0.230 ± 0.025, 95% CI: −0.279 to −0.181).

##### 3.2.5.4. BMI

For BMI, the Insomnia –> Physical activity path was associated with a significant group difference (*p* = 0.004) (non-obese: −0.100 ± 0.015; *p* < 0.001; obese: −0.015 ± 0.025, *p* = 0.563). Thus, the path in the obese patients was not significant compared to the path in the non-obese patients.

##### 3.2.5.5. Spatial extent of pain (PRI)

When comparing those with lower and higher PRI, we noted three significant group differences. Thus, more widespread pain in the body was associated with stronger effects for these three paths:

(1) Insomnia –> Pain intensity (group difference: *p* = 0.030; lower PRI: 0.199 ± 0.017 *p* < 0.001 vs. higher PRI: 0.253 ± 0.018 *p* < 0.001);
(2) Insomnia –> Catastrophizing (group difference: *p* = 0.003; lower PRI: 0.317 ± 0.016 *p* < 0.001 vs. higher PRI: 0.381 ± 0.015 *p* < 0.001); and
(3) Insomnia –> Fear avoidance (group difference: *p* = 0.013; lower PRI: 0.223 ± 0.017 *p* < 0.001 vs. higher PRI: 0.271 ± 0.017 *p* < 0.001).

## 4. Discussion

This large cohort study (*N* > 6,400) of patients with an insomnia prevalence of more than 60% (62.3%) produced several important results when the paths from insomnia to pain intensity were explored. Both direct and indirect (mediating) paths existed for the insomnia–pain intensity relationship. The mediating effects taken together were weaker than the direct effect between insomnia and pain intensity. Three of the five mediating paths were significant and associated with small effect sizes; the mediating effects via Catastrophizing and Acceptance showed the strongest and equal mediating paths, which were followed by those via Fear avoidance. Insomnia showed direct significant correlations with Psychological distress, Catastrophizing, Acceptance, and Fear avoidance stronger than those with Pain intensity. The five moderators did not significantly affect the mediating paths.

### 4.1. A high prevalence of insomnia

A high prevalence of insomnia (62.3%) was observed in this cohort of chronic pain patients, which was very similar to the figures reported from smaller SQRP cohorts—i.e., 65%–66% (9, 69). A recent SR reported an insomnia prevalence of 72.9% in chronic pain patients using ISI (8); however, a similar prevalence (75.3%) was noted for studies using the Pittsburgh Sleep Quality Index (PSQI). Hence, these figures are somewhat higher than the present prevalence, but this cohort is markedly larger than the studies included in the SR (ISI, total *N* = 2,578; PSQI, total *N* = 3,597) (8). Although the prevalence rates differ somewhat, most studies have indicated that insomnia probably affects most patients with chronic pain. In addition to pain, insomnia, poor sleep, and sleep deprivation are associated with conditions, such as cancer, type 2 diabetes, hypertension, heart disease, reduced cognitive functioning, somatic complaints, psychological distress, fatigue, and impaired quality of life (70–75). As such, sleep appears to be very important in the regulation of biological processes pivotal for health (71). The present cross-sectional study indeed confirmed a significant direct path between reported Insomnia and Pain intensity. Hence, it seems highly reasonable to include screening for sleep problems in the assessment of chronic pain patients (5).

### 4.2. Mediating effects

Insomnia was significantly related to Pain intensity (and vice versa) both directly and indirectly via mediating paths. The direct effect was stronger than the five mediating effects taken together. Together, the direct and indirect effects explained approximately 25% of the variation in the latent variable Pain intensity (*R*^2 ^= 0.254) (Figure 2). Three of the five investigated mediating paths were significant and had small effect sizes. The strongest mediating effects were noted for Catastrophizing and Acceptance, which had equal mediating effects (both *β* = 0.053). Thus, some of the effects of Insomnia on Pain intensity were indirect and thus mediated by Catastrophizing and Acceptance. Fear avoidance was also a mediator, although its role (*β* = 0.016) was somewhat weaker than that of Catastrophizing and Acceptance. The mediating path via Psychological distress was not significant, and the mediating path via Physical activity was associated with a non-significant effect size.

*Catastrophizing* is the tendency to amplify negative cognitive and emotional processes related to pain. It is mainly due to environmental factors although heritability factors also exist (36%–37%) (76, 77). Catastrophizing negatively influences pain sensation and intensity (78, 79), depression and anxiety (79), and disability and quality of life (79) and may be a risk factor for chronic pain development (79, 80). Catastrophizing may be a transdiagnostic process that ties pain and depression/emotion together (81–83). It is associated with worse treatment outcomes, but it is modifiable and therefore a possible prevention and treatment target (84, 85). Several explanations exist for the role that catastrophizing might have in chronic pain as briefly summarized by Racine et al. (85). These explanations posit that catastrophizing not only reflects pain but also has a causal influence on pain and its consequences (85). Consistent with the above literature, we noted that both parts of the mediating path via Catastrophizing had positive *β* values—i.e., the path from Insomnia to Pain intensity increased pain intensity. Our results are in line with a small study of fibromyalgia patients who identified pain helplessness (cf. PCS-help) as a significant mediating path between insomnia and pain intensity (86).

*Fear avoidance* is a coping strategy that involves avoidance of physical and social activities of daily living due to fear of increased pain and/or fear of injury or reinjury (53). This is associated with risk of pain chronification, increased chronic pain intensity, and disability (87–89). Catastrophizing could be a key driver in the fear avoidance model of pain (79). Similarly, the mediating path via Fear avoidance was also positive (i.e., insomnia increased pain intensity via this path); however, the mediating path was weaker than the mediating paths via catastrophizing and acceptance. Even when these two mediating paths (i.e., via Fear avoidance and Catastrophizing) are combined, the effect size remains small. Several instruments are available for measuring fear avoidance (87), and TSK is one of those. Although these instruments have been extensively used, there are still some psychometric critiques (87). For example, TSK contains items concerning fear but only in the context of injury or reinjury (87), which may help explain the lower but still significant coefficient for this mediating path. A broader and more general measure of fear avoidance is desirable in future studies and may be associated with a more valid picture of its role in the insomnia–pain intensity relationship.

The mediating effect of *Acceptance* on the Insomnia–Pain intensity relationship was associated with lower pain intensity in contrast to the mediating result via catastrophizing (i.e., both parts of this mediating path had negative *β* values). Acceptance, a component of psychological flexibility, is the willingness to remain in contact with and to actively engage in unpleasant experiences despite chronic pain (90–92). Low acceptance has been perceived as an unproductive inner struggle with the pain experience, including attempts to avoid pain. Low acceptance is associated with chronic pain management problems and with suffering, depressive symptoms, avoidance, healthcare utilization, and poorer functioning (59, 93–97). Thus, patients reporting higher levels of acceptance may struggle less while trying to control and fall asleep and have fewer fears of being tired or exhausted during the day. Increasing evidence has emerged with regard to the effect of acceptance and commitment therapy (ACT) on primary and comorbid insomnia (98) and chronic pain conditions (99). Chronic pain conditions are associated with abnormal hyper-connectivity of brain networks associated with self-reflection (default mode, DMN), emotion (salience, SN), and cognitive control (frontal-parietal, FPN) (100). Mainly consistent with a few other studies, reductions within and between these networks were noted after ACT treatment (100).

Longitudinal studies indicated that depressive and/or anxiety symptoms mediated the effect between insomnia and pain symptoms (17). The mediating path via *Psychological distress* was not significant in this cross-sectional study. Other cross-sectional studies indicated that anxiety and/or depressive symptoms were positive mediators as summarized in the referred SR (43). In this context, more complex models have been called for (43). Our study is a parallel mediating study—i.e., the mediating latent variables competed against each other. Except for Physical activity, all mediating latent variables were intercorrelated according to a principal component analysis in this sample (data not shown). Although psychological distress aspects may be significant in a single mediation analysis, other psychological factors (e.g., catastrophizing, fear avoidance, and acceptance) may be more important in a parallel mediation analysis.

A reduced prevalence of insomnia was found in physically active individuals with chronic pain (37). However, although the mediating path via *Physical activity* level was significant, the importance from a clinical perspective was negligible. Our results with regard to physical activity were consistent with those of a longitudinal study of young adults in a population study (101).

### 4.3. Moderation aspects

*Sex* was not a moderator of the paths explored even though women report, for example, higher prevalence of chronic pain and widespread pain (102–104), greater pain severity (not consistent) (46, 48, 105–107), and higher prevalence of sleep problems/insomnia (108–110).

None of the moderators significantly affected the mediating paths considered in this study. However, for moderators other than sex, some of the direct effects of insomnia showed group differences, which may have clinical implications.

*Age* was a significant moderator of the direct effects between Insomnia and several of the latent variables. In older patients, three of the associations with Insomnia were stronger (Catastrophizing, Fear avoidance, and Psychological distress) and one weaker (Pain intensity). Thus, in older ages, insomnia will be more strongly tied to psychological distress and two negative coping aspects.

*Education level* not only reflects school background but also serves as a proxy for socioeconomic status, including work situation. The prevalence of chronic pain, severity of pain, and disability are inversely related to socioeconomic status (111–114). The only difference was found for the Acceptance–Pain intensity relationship: a stronger negative coefficient was noted for those with a university education. This moderating effect must be confirmed in other studies before making any conclusions as to whether the design of treatments needs to vary by education level.

Some studies reported that both pain and insomnia are related to *obesity* (20, 21), but the relationships between obesity/BMI and insomnia symptoms/disorders have been challenged in recent meta-analyses (37, 75, 115). The clinical importance of the fact that BMI significantly moderated the path from Insomnia to Physical activity remains unclear.

Positive associations exist between insomnia and the *spatial extent of pain* in the body both in cross-sectional and longitudinal perspectives (11, 15, 116). Both Norwegian and Swedish longitudinal studies reported that insomnia is a risk factor for increased spreading of pain (15, 116). This study shows that more widespread pain (an increased spatial extent) was associated with stronger effects for three of the direct paths from Insomnia—i.e., to Pain intensity, Catastrophizing, and Fear avoidance. Consistent with our results, a recent network study (insomnia was not included) reported that the pain extent was a moderator of the relationships between several of the variables included in the present PLS-SEM (117).

### 4.4. Insomnia correlations (i.e., the direct paths from Insomnia)

The importance of assessing sleep problems, especially insomnia, is further strengthened by the present observations that insomnia showed significant associations (i.e., significant direct path coefficients) with all clinical aspects of the clinical presentation included in this study (Figure 2 and Table 4). Thus, Insomnia was most strongly associated with Psychological distress (positively), Catastrophizing (positively), and Acceptance (negatively). The paths with Pain intensity and Fear avoidance were positive and significant but weaker than for the other latent variables. On a general level, several other studies have reported associations between insomnia and these clinical variables (9, 37, 118–120). However, our results are partly in contrast to a network study that demonstrated that insomnia had a stronger correlation with pain intensity aspects than with depression and anxiety symptoms (121). On the other hand, our results agree with another smaller SQRP real-world study from one department, which found that ISI had the strongest correlations with anxiety and depressive symptoms (9). Obviously, more studies are required to understand these intercorrelations, including both their causal cross-sectional and longitudinal associations.

### 4.5. Latent variables correlating with pain intensity (i.e., the direct paths to pain intensity)

One important observation is that the correlations (direct paths) between Insomnia and the five mediators are stronger than the correlations between the five mediators and pain intensity. Nevertheless, our results concerning the direct paths from the five mediators to pain intensity indicate that it is clinically important that the assessment process focuses on more than just psychological distress levels. In fact, it can be even more important to focus on insomnia and coping aspects such as catastrophizing and acceptance. Hence, Pain intensity had the strongest absolute correlation (positive *β*) with Insomnia followed by Acceptance (negative *β*) and Catastrophizing (positive *β*). Interestingly, the Psychological distress–Pain intensity direct path did not reach significance (*p* = 0.054), and other studies have reported weak intercorrelations between pain intensity and psychological distress aspects (117, 121). Such results concerning the levels of these two latent variables do not invalidate that psychological distress is a common comorbidity in chronic pain (122, 123).

### 4.6. Clinical significance

The present study confirmed a significant direct path not only between Insomnia and Pain intensity (and vice versa) but also via other mediating latent variables—i.e., Catastrophizing, Acceptance, and Fear avoidance. In addition, Insomnia showed even stronger direct associations with Psychological distress, Catastrophizing, and Acceptance. As all these clinical facets contribute to considerable suffering for the individual and are interconnected both at the psychological and physiological level, it is relevant to address them in a clinical setting. Poor acceptance, for example, is characterized by an inability to come to terms with the chronic pain condition where considerable effort is made to solve an unsolvable problem, leaving patients in constant limbo, further contributing to their stress. The fear and helplessness of catastrophizing will naturally also contribute to negative behavioral feedback loops, which produce considerable stress and heightened pain. Poor sleep, psychological distress, catastrophizing, and low acceptance all constitute considerable stressors that can affect the balance that individuals maintain between biological, psychological, and sociocontextual aspects or factors, leading to a vicious unhealthy cycle. For example, Haack et al. (124) found that sleep deficiency can affect various neurobiological systems that influence nociceptive processing. Hence, it is easy to perceive how such a situation impacts, for example, autonomous control, leading to a restless state and poorer sleep. As such, vicious psychological and physiological circles are intertwined, which is both problematic and hopeful in the sense that identifying and influencing one factor might lead to beneficial effects for the whole circle. Clinical assessments including both pain and insomnia are reasonable since both direct and mediating factors are present between insomnia and pain intensity. Although Swedish IPRPs generally address acceptance and behaviors such as avoidance, they do not for the most part adequately address insomnia (125). This oversight neglects a huge potential to attend to an important physiological key factor that can have many beneficial effects. Future research should focus on illuminating how sleep interventions influence pain intensity and other important key factors contributing to distress for chronic pain patients.

### 4.7. Strength and limitations

A strength of this study is its large cohort of chronic pain patients with nationwide representation. Our results are relevant for patients referred to specialist care, which represent the most complex patients with chronic pain on a general level. Most of the mediating studies included in the SR of mediating paths were single mediation studies (43). Hence, they represent a too simplistic view of the clinical presentation of patients with chronic pain. Parallel mediation more adequately mirrors the clinical situation. Several of the latent variables had several indicators, which is an advantage from a measurement error point of view compared to path analyses, which only use single items representing a certain latent variable. The present cohort is representative—with respect to sex and education level—for the chronic pain patients referred to specialist departments in Sweden. The moderator analyses showed no major differences in path patterns. Hence, we therefore have no reason to believe that a more even distribution regarding, e.g., sex or education level would change our results.

Another strength of this study is the consistency of the overall findings supports the conclusions. Similarly, the overall point—to quantify the relative contributions of these latent variables on the insomnia/ chronic pain cycle—does not depend entirely on many individual statistical tests that are vulnerable to false positives.

The obvious limitation is that our study is based on cross-sectional data. Thus, future studies using PLS-SEM may include within-day and day-to-day variability studies. On the other hand, from the perspective of the clinical assessment, it is also important that cross-sectional studies deepen the understanding of how insomnia and pain intensity interact. Our PLS-SEM analysis is based on a specific overarching hypothesis concerning the insomnia–pain intensity relationship, which in essence was investigated in the SR of Whibley et al. (43). Future studies—which may be based on other hypotheses—are needed to validate our results. Moreover, cohort heterogeneity concerning the latent variables (Insomnia–Pain intensity relationship including mediators) may be present for other aspects than the moderators investigated in this study. Moreover, questionnaires such as ISI serve as a proxy for capturing insomnia problems; polysomnography is considered the golden standard, and actigraphy is considered the second best way to capture insomnia problems (126, 127). Measuring insomnia and other sleep problems with more objective methods may further strengthen results from PLS-SEM analyses. The inclusion of objective methods has the disadvantage that the studies must be considerably smaller both for practical and financial reasons.

### 4.8. Conclusions

This large cohort study of chronic pain patients with an insomnia prevalence of more than 60% (62.3%) revealed that both direct and mediating paths exist for the Insomnia–Pain intensity relationship. Together, these mediating effects were weaker than the direct effect between Insomnia and Pain intensity. The mediating effects via Catastrophizing and Acceptance showed the strongest and equal mediating paths, which were followed by those via Fear avoidance. Insomnia showed direct significant correlations with Psychological distress, Catastrophizing, Acceptance, and Fear avoidance stronger than those with Pain intensity. The five moderators did not significantly affect the mediating paths. Future research should focus on illuminating how sleep interventions influence pain intensity and other important key factors that contribute to the distress of chronic pain patients.

## Data Availability

The raw data supporting the conclusions of this article will be made available by the authors, without undue reservation.
