# Supplementary material for: Catastrophizing and acceptance are mediators between insomnia and pain intensity—an SQRP study of more than 6,400 patients with non-malignant chronic pain conditions
Source: Front Pain Res (Lausanne). 2023 Sep 27;4:1244606. doi: 10.3389/fpain.2023.1244606 (PMC10565667; doi:10.3389/fpain.2023.1244606)
Supplement: Supplementary file 1 [file Datasheet1.docx]

Supplementary Tables

Catastrophizing and acceptance are mediators between insomnia and pain intensity – a SQRP study of more than 6400 patients with chronic non-malignant pain conditions

Björn Gerdle*, Elena Dragioti, Marcelo Rivano Fischer, Huan-Ji Dong, Åsa Ringqvist

*** Correspondence: Björn Gerdle** [bjorn.gerdle@liu.se](mailto:bjorn.gerdle@liu.se)

**Supplementary Table 1**. Internal consistency reliability and Convergent validity (only relevant for constructs with more than one indicator).

|  | **Internal consistency  reliability** | **Convergent  validity** |
| --- | --- | --- |
| **Latent variables** | Rho-c | AVE |
| Pain intensity | 0.910 | 0.772 |
| Catastrophizing | 0.906 | 0.763 |
| Psychological distress | 0.925 | 0.755 |

**Supplementary Table 2**. Discriminant validity according to Heterotrait-monotrait ratio (HTMT).

| **Relationships** | **HTMT** |
| --- | --- |
| Catastrophizing ↔ Acceptance | 0.576 |
| Fear-avoidance ↔ Acceptance | 0.481 |
| Fear-avoidance ↔ Catastrophizing | 0.596 |
| Insomnia ↔ Acceptance | 0.305 |
| Insomnia ↔ Catastrophizing | 0.371 |
| Insomnia ↔ Fear-avoidance | 0.244 |
| Pain intensity ↔ Acceptance | 0.408 |
| Pain intensity ↔ Catastrophizing | 0.433 |
| Pain intensity ↔ Fear-avoidance | 0.337 |
| Pain intensity ↔ Insomnia | 0.410 |
| Physical activity ↔ Acceptance | 0.195 |
| Physical activity ↔ Catastrophizing | 0.060 |
| Physical activity ↔ Fear-avoidance | 0.196 |
| Physical activity ↔ Insomnia | 0.086 |
| Physical activity ↔ Pain intensity | 0.153 |
| Psychological distress ↔ Acceptance | 0.470 |
| Psychological distress ↔ Catastrophizing | 0.639 |
| Psychological distress ↔ Fear-avoidance | 0.361 |
| Psychological distress ↔ Insomnia | 0.490 |
| Psychological distress ↔ Pain intensity | 0.370 |
| Psychological distress ↔ Physical activity | 0.096 |

**Supplementary Table 3**. Variance inflation factor (VIF) values.

| **Indicators** | **VIF** |
| --- | --- |
| ISI | 1.000 |
| NRS-7d | 2.594 |
| MPI-Pain severity | 2.778 |
| RAND36-bodily pain-rev | 1.701 |
| PCS-help | 2.303 |
| PCS-magn | 1.913 |
| PCS-rum | 2.016 |
| TSK | 1.000 |
| Exercise | 1.000 |
| CPAQ8-tot | 1.000 |
| HADS-tot | 3.429 |
| MPI-distress | 2.495 |
| RAND36-mental health-rev | 3.656 |
| RAND36-role emotional-rev | 1.679 |

-rev=the variable was revised to indicate a troublesome situation. * indicates that the variable not was included in the final PLS-SEM analysis
NRS-7d=Pain intensity according to a numeric rating scale; HAD=The Hospital Anxiety and Depression Scale; HAD-tot=sum of the two subscales of HAD; MPI=Multidimensional Pain Inventory; rand36=the free version of the Short Form Health Survey (sf36); TSK=Tampa Scale for Kinesiophobia; CPAQ8-tot=Chronic Pain Acceptance Questionnaire 8-item version- total score; ISI=Insomnia Severity Index; PCS=Pain Catastrophizing Scale; PCS-rum=rumination subscale of PCS; PCS-help=helplessness subscale of PCS; PCS-Magn=magnification subscale of PCS.

**Supplementary Table 4**. Predictive power (*Q²predict)*.

| **Prediction summary** | **Q²predict** |
| --- | --- |
| Acceptance | 0.093 |
| Catastrophizing | 0.120 |
| Fear-avoidance | 0.060 |
| Pain intensity | 0.143 |
| Physical activity | 0.007 |
| Psychological distress | 0.216 |

**
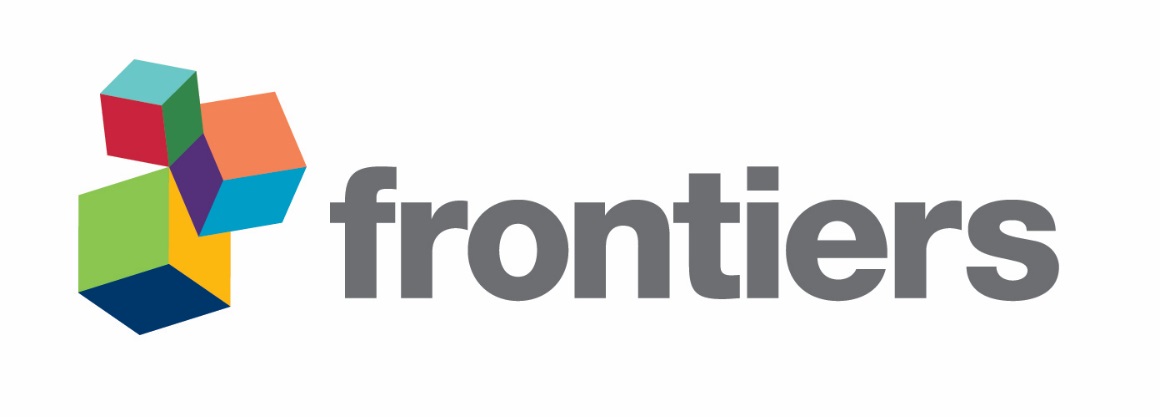
**
